# Assessing the Impact of Extended Lovenox Prophylaxis on Outcomes Following Postmastectomy Microsurgical Breast Reconstruction

**DOI:** 10.1093/asjof/ojag138.003

**Published:** 2026-07-24

**Authors:** Sajni Parikh, Natalia Mejia, Carolyn Silverman, Gabrielle Knauer, Aryan Gupta, Gabriel De La Cruz, Adam Walchak, Sameer Patel

**Affiliations:** Division of Plastic and Reconstructive Surgery, Temple University Hospital, Philadelphia, PA; Division of Plastic and Reconstructive Surgery, Fox Chase Cancer Center, Philadelphia, PA; Lewis Katz School of Medicine, Philadelphia, PA; Lewis Katz School of Medicine, Philadelphia, PA; Lewis Katz School of Medicine, Philadelphia, PA; Division of Plastic and Reconstructive Surgery, Temple University Hospital, Philadelphia, PA; Division of Plastic and Reconstructive Surgery, Fox Chase Cancer Center, Philadelphia, PA; Division of Plastic and Reconstructive Surgery, Fox Chase Cancer Center, Philadelphia, PA

## Abstract

**Purpose:**

Venous thromboembolism (VTE) causes mortality in 100,000 patients annually in the United States. The ACCP guidelines proposed giving extended chemoprophylaxis to patients who had a high VTE risk (Caprini score ≥5) undergoing abdominal or pelvic surgery for cancer. However, postmastectomy microsurgical breast free reconstruction was excluded from these recommendations. We aim to compare VTE outcomes and procedure related complications between short and extended postoperative chemoprophylaxis. Methods: A retrospective chart review was performed of patients from our institution who underwent DIEP flap reconstruction and were discharged on either short-term (2 weeks) enoxaparin from January 2017- June 2025. Those with a history of prior VTE, coagulopathy, or anticoagulation use were excluded. Chi-squared analyses independently compared PE and DVT rates before and after the implementation of extended postoperative prophylaxis.

**Methods:**

A retrospective chart review was performed of patients from our institution who underwent DIEP flap reconstruction and were discharged on either short-term (2 weeks) enoxaparin from January 2017- June 2025. Those with a history of prior VTE, coagulopathy, or anticoagulation use were excluded. Chi-squared analyses independently compared PE and DVT rates before and after the implementation of extended postoperative prophylaxis.

**Results:**

A total of 358 patients were included: 62 received short-term prophylaxis and 296 received extended prophylaxis, both cohorts received enoxaparin 40 mg daily. DVT and PE rates were 2.48% and 4.9% in the short-term prophylaxis cohort, compared with 1.87% and 0% with extended prophylaxis. Extended prophylaxis was associated with significantly lower rates of PE and DVT (P = 0.04 and P = 0.002, respectively); there was no significant difference in hematoma rates between cohorts (P = 0.76).

**Conclusion:**

The implementation of extended postoperative chemoprophylaxis was associated with significant reductions in the rates of both DVT and PE, without an increased bleeding risk. Our findings support integration of the described regimen into standardized postoperative prophylaxis protocols.

